# Validation of a Clinical Instrument for Measuring the Severity of Acute Bronchitis in Children – The BSS-ped

**DOI:** 10.2174/1874306401812010081

**Published:** 2018-12-19

**Authors:** Siegfried Lehrl, Peter Kardos, Heinrich Matthys, Wolfgang Kamin

**Affiliations:** 1 Department of Psychiatry and Psychotherapy, Friedrich-Alexander University Erlangen-Nuremberg, Erlangen, Germany; 2 Group Practice and Centre for Pneumology, Center for Respiratory, Allergy and Sleep Medicine at Red Cross Maingau Hospital, Frankfurt am Main, Germany; 3 Department of Pneumology, University Hospital Freiburg, Freiburg, Germany; 4 Clinic for Paediatrics, Evangelic Hospital Hamm, Hamm, Germany

## Validation of a Clinical Instrument for Measuring the Severity of Acute Bronchitis in Children – The BSS-ped

The Open Respiratory Medicine Journal, **2018**, 12: 50-60

**Figure 2 has been revised as follows:**


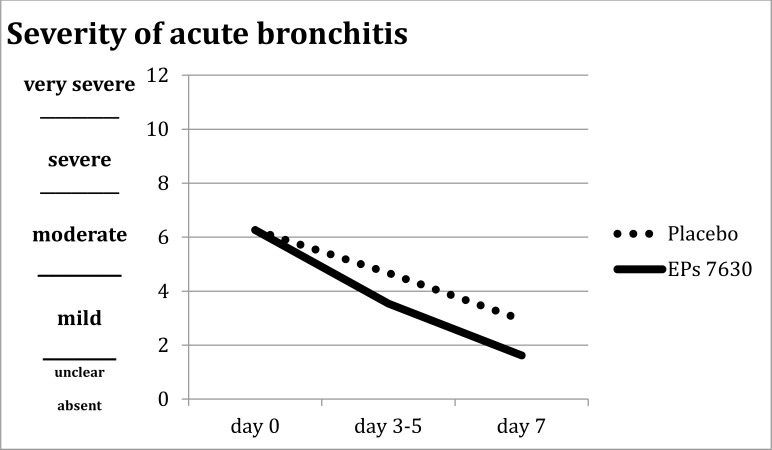


**The original Figure 2 provided was:**


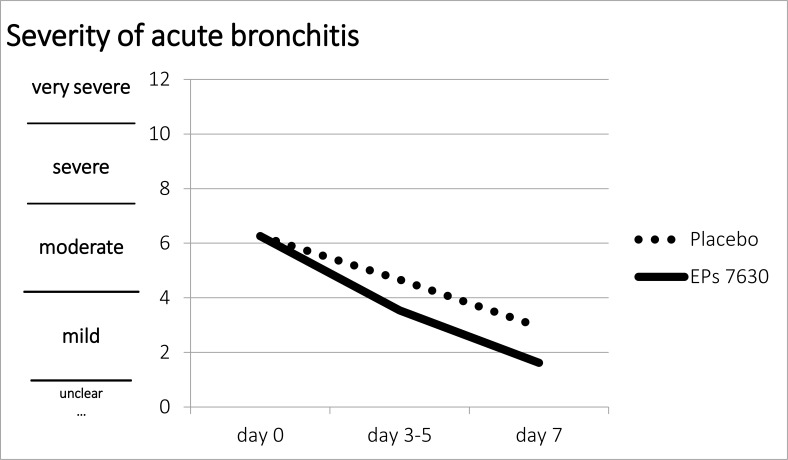


**A foot note has been added in Table 2 as follows:**

° pulmonary rales at auscultation

